# Evaluation of ^11^C-choline PET/CT for primary diagnosis and staging of urothelial carcinoma of the upper urinary tract: a pilot study

**DOI:** 10.1007/s00259-014-2871-y

**Published:** 2014-08-08

**Authors:** Naoto Sassa, Katsuhiko Kato, Shinji Abe, Shingo Iwano, Shinji Ito, Mitsuru Ikeda, Kazuhiro Shimamoto, Seiichi Yamamoto, Tokunori Yamamoto, Momokazu Gotoh, Shinji Naganawa

**Affiliations:** 1 Department of Urology, Nagoya University Graduate School of Medicine, Nagoya, Japan; 2 Department of Radiological and Medical Laboratory Sciences, Nagoya University Graduate School of Medicine, 1-20 Daikominami 1-chome, Higashi-ku, Nagoya, 461-8673 Japan; 3 Department of Radiological Technology, Nagoya University Hospital, Nagoya, Japan; 4 Department of Radiology, Nagoya University Graduate School of Medicine, Nagoya, Japan

**Keywords:** ^11^C-Choline PET/CT, Urothelial carcinoma, Upper urinary tract, Renal pelvis, Ureter

## Abstract

**Purpose:**

We conducted a pilot study to prospectively evaluate the efficacy of PET/CT with ^11^C-choline (choline PET/CT) for primary diagnosis and staging of urothelial carcinoma of the upper urinary tract (UUT-UC).

**Methods:**

Enrolled in this study were 16 patients (9 men, 7 women; age range 51 – 83 years, mean ± SD 69 ± 10.8 years) with suspected UUT-UC. The patients were examined by choline PET/CT, and 13 underwent laparoscopic nephroureterectomy and partial cystectomy. Lymphadenectomy and chemotherapy were also performed as necessary in some of the patients. Of the 16 patients, 12 were confirmed to have UUT-UC (7 renal pelvis carcinoma and 5 ureteral carcinoma), 1 had malignant lymphoma (ureter), 1 had IgG4-related disease (ureter), and 2 had other benign diseases (ureter).

**Results:**

Of the 16 study patients, 13 showed definite choline uptake in urothelial lesions, and of these, 11 had UUT-UC, 1 had malignant lymphoma, and 1 had IgG4-related disease. Three patients without choline uptake comprised one with UUT-UC and two with benign diseases. Of the 12 patients with UUT-UC, 3 had distant metastases, 2 had metastases only in the regional lymph nodes, and 7 had no metastases. Distant metastases and metastases in the regional lymph nodes showed definite choline uptake. The outcome in patients with UUT-UC, which was evaluated 592 – 1,530 days after surgery, corresponded to the patient classification based on the presence or absence of metastases and locoregional or distant metastases. Choline uptake determined as SUVmax 10 min after administration was significantly higher than at 20 min in metastatic tumours of UUT-UC (*p* < 0.05), whereas there was no statistically significant difference between the SUVmax values at 10 and those at 20 min in primary tumours of UUT-UC.

**Conclusion:**

This study suggests that choline PET/CT is a promising tool for the primary diagnosis and staging of UUT-UC.

## Introduction

Urothelial carcinoma of the upper urinary tract (renal pelvis and ureter; UUT-UC) is an infrequent genitourinary malignancy accounting for 5 % of urothelial cancers and less than 10 % of renal tumours [1]. Analysis of epidemiological and survival patterns of UUT-UC over the past 30 years in a review of a large, population-based database in the USA has shown that the incidence of UUT-UC has slowly risen, and that increasing patient age, male gender, Afro-American race, bilateral UUT-UC and regional/distant disease are all associated with poorer survival [2].

For the primary diagnosis and staging of UUT-UC, radiographic imaging by intravenous and retrograde pyelography and CT scanning, ureteroscopic visualization and biopsy of the tumour, and urine cytology are usually performed. Although ^18^F-FDG is the radiopharmaceutical most frequently used in PET, FDG is not a useful tracer for the detection of primary tumours of the urinary tract because of its renal excretion. Therefore, the utility of FDG PET in the detection of urinary tract tumours is limited to distant metastases [3, 4]. It has been reported that FDG PET/CT is superior to conventional evaluations in detecting occult metastases in patients with invasive bladder cancer [5, 6]. However, another study has shown no advantage of FDG PET/CT over CT alone for lymph node staging in invasive bladder cancer [7].

A number of studies have demonstrated that PET/CT with ^11^C-choline (choline PET/CT) is useful in the detection of lymph node metastases of prostate cancer [8–15] and also the detection of primary prostate cancer with certain limitations [16–20]. However, to the best of our knowledge, there have been no studies on the efficacy of choline PET/CT in the initial diagnosis and local staging of UUT-UC. The purpose of this pilot study was to prospectively evaluate the impact of choline PET/CT in localizing the primary tumour and staging of UUT-UC. In this study, the effects of the choline PET/CT findings on the clinical management of UUT-UC and the outcomes in patients with UUT-UC were also evaluated.

## Materials and methods

### Patients

All procedures followed the clinical guidelines of our hospital and were approved by the institutional review board. A complete description of the study was given to all participating patients and a consent form was signed by the patients. We enrolled 16 consecutive patients with a clinical suspicion of UUT-UC (9 men, 7 women; age range 51 – 83 years, mean ± SD 69 ± 10.8 years) who were referred to the Department of Urology of our hospital between March 2008 and January 2011. All patients were examined using choline PET/CT 3 – 65 days (mean 19.2 days) before surgery. The patients were classified clinically according to the 2009 TNM staging system [21]. Two patients were inoperable and one patient needed no surgery; all remaining patients underwent laparoscopic nephroureterectomy together with partial cystectomy (LNUx). In the patients who were diagnosed with lymph node metastases, lymphadenectomy was performed. In addition, after choline PET/CT some of the patients received neoadjuvant chemotherapy with gemcitabine and cisplatin [22, 23] and chemotherapy with methotrexate, vinblastine, doxorubicin and cisplatin, with gemcitabine and carboplatin, and with gemcitabine .

### Choline PET/CT image acquisition

PET and CT imaging was performed using a combined PET/CT system (Biograph Sensation 16; Siemens Medical Solutions, Forchheim, Germany, and Hoffman Estates, IL). The system combines a full-ring PET scanner equipped with lutetium orthosilicate crystal detectors and a 16-slice high-resolution spiral CT scanner. All patients were examined after fasting for 6 h. Choline PET/CT was performed 10 and 20 min after intravenous injection of 3.7 MBq/kg of ^11^C-choline. In the PET/CT scanner, the patients were positioned head first and supine. Their arms were down and their hands were clasped on the abdomen throughout the scanning procedure rather than elevated above the head. This position was chosen in consideration of patient comfort. First, a topogram was acquired to define the imaging range for both CT and PET. The range was typically chosen to extend from the scull base to the mid-thigh level. A single nonenhanced continuous spiral multislice CT scan was performed (120 kVp tube voltage, 100 mAs effective tube current, 16 × 1.5-mm detector configuration, gantry rotation time 420 ms, 30-mm table feed per rotation). The dose was adjusted to the actual level of attenuation. For this purpose, attenuation was measured during the first half of helical rotation, and the effective tube current was automatically modulated for the second half (CARE Dose; Siemens Medical Solutions, Hoffman Estates, IL). Data were acquired for 1 min per bed position following the CT scan. In all patients scanning was started caudally and progressed cranially. The mean radiation doses used in this study were 1.1 mSv in choline PET and 7.05 mSv in CT.

CT and PET images were reconstructed using standard clinical protocols. CT images with a matrix size of 512 × 512 and a slice thickness of 3 mm with a 1.5-mm increment were generated using filtered back-projection. PET images with a matrix size of 128 × 128 were reconstructed using a 3-D numerical reconstruction algorithm (e.soft; Siemens Medical Solutions). CT-derived attenuation maps were used for PET attenuation correction.

### PET/CT data analysis

For visual and quantitative analysis, images were displayed on a clinical image analysis workstation that allowed interactive exploration of CT, PET and fused PET/CT image data. The loci where choline uptake was visibly higher than the activity of adjacent areas were tentatively considered uptake-positive. For each uptake-positive locus the intensity of uptake was further analysed semiquantitatively. For this purpose the coronal slice that contained the maximum uptake was selected and the maximum standardized uptake value (SUVmax) with regard to body weight was determined. In this study, it was demonstrated that, when SUVmax was lower than 2.2, the contour and extent of the uptake area were vague. Therefore, SUVmax 2.2 was considered negative uptake. The size of tumours (long diameter × short diameter × height) was measured on CT images.

### Histopathological typing

Histopathological examinations were performed in 14 of the 16 study patients. In 13 patients specimens were obtained from tissue resected during LNUx and in 1 patient from tissue obtained at autopsy. Histological grades were classified according to the World Health Organization classification [24].

### Statistical analysis

Kaplan-Meier analysis was used to compare the survival of the patients with UUT-UC in relation to the presence and location of metastases. The analyses were performed with the log-rank test using SPSS v. 20 (IBM, Armonk, NY). The SUVmax at 10 and 20 min after injection were compared using the paired *t*-test. A value of *p* <0.05 was considered significant.

## Results

The profiles of the 16 study patients, and the choline uptake determined as SUVmax and the sizes estimated on CT images of the primary tumours are shown in Table 1. The clinical findings, treatment, and outcomes in the patients, and histopathological findings of the primary tumours are shown in Table 2.

**Table 1:** Patient profiles and ^11^C-choline uptake and sizes of primary tumours estimated on PET/CT images

Patient no.	Age (years)	Sex	Primary tumour				
			Side	Location	^11^C-Choline uptake (SUVmax)		Size (mm)^a^
					10 min	20 min	
1	61	M	Right	Renal pelvis	2.48	3.79	29 × 25 × 20
2	67	F	Right	Renal pelvis	5.52	4.36	37 × 26 × 20
3	50	F	Left	Ureter	2.80	2.94	10 × 9 × 12
4	73	F	Right	Ureter	<2.2	<2.2	Indeterminable^b^
5	56	M	Right	Ureter	<2.2	<2.2	15 × 15 × 15^b^
6	81	M	Right	Renal pelvis	3.83	3.43	19 × 25 × 12
7	83	M	Right	Renal pelvis	2.74	2.83	10 × 9 × 12
8	81	M	Left	Renal pelvis	2.31	3.22	8 × 8 × 15
9	69	F	Left	Renal pelvis	4.06	3.97	23 × 20 × 12
10	75	M	Right	Ureter	2.49	2.18	10 × 9 × 8
11	64	F	Right	Ureter	6.09	6.28	11 × 10 × 8
12	53	M	Right	Renal pelvis	7.85	8.08	46 × 45 × 40
13	56	F	Right	Ureter	3.25	2.64	12 × 11 × 12
14	81	M	Left	Ureter	3.97	3.79	10 × 10 × 10
15	67	F	Right	Ureter	<2.2	<2.2	14 × 14 × 50
16	81	M	Right	Ureter	6.59	3.80	25 × 17 × 25

^a^Long diameter × short diameter × height

^b^Nonmalignancy

**Table 2:** Clinical findings, treatment, and outcomes in patients and histopathological findings of tumours

Patient no.	TNM classification^a^	Treatment	Interval from PET/CT to LNUx (days)	Histopathological findings	Outcome	Days after LNUx^b^
1	III (T3N0M0)	LNUx	33	UC, G2 > G3 pT3, ly0, v0, n0	NED	1,530
2	IV (T4N2M1)	Inoperable	–	UC, G3, pT4	Died from cancer	25 (after PET/CT )
3	I (T1N0M0)	LNUx	45	Malignant lymphoma	NED	1,560
4	–	LNUx	10	No malignancy	NED	699
5	–	–	–	–	NED	–
6	0a (T1aN0M0)	LNUx	14	UC, G1, pTa, ly0, n0, v0	Died from cancer	725
7	I (T1N0M0)	LNUx	7	UC, G2, pT1, ly0, n0, v0	NED	1,451
8	I (T1N0M0)	LNUx	13	UC, G2, pT1, ly0, n0, v0	NED	1,426
9	II (T2N0M0)	LNUx	42	UC, G2 = G3, pT2, ly0, v0, n0	NED	1,402
10	I (T1N0M0)	LNUx	45	UC, G2 > G3, pT1, ly0, v0, n0	NED	1,399
11	IV (T2N1M0)	NAC + LNUx + MVAC	4	UC, G2-3, pT2, ly+, v+, n+	NED	1,414
12	IV (T4N0M1)	LNUx + MVAC	65	UC > SCC, G3, pT4	Died from cancer	180
13	–	LNUx	3	IgG4-related disease	NED	743
14	IV (T3N1M0)	LNUx + (G + CBDCA)	3	UC, G3, pT3, INFβ, ew0, ly0, n0	Died from cancer	600
15^c^	IV (T4N0M0)	LNUx + NAC	61	UC, G3, pT4, INFβ, ly2, v1,n0	Alive with cancer	592
16	IV (T3N2M1)	Inoperable, G	–	–	Alive with cancer	510 (after PET/CT)

*LNUx* laparoscopic nephroureterectomy and partial cystectomy; *NAC* neoadjuvant chemotherapy with gemcitabine and cisplatin (two cycles) before or after LNUx; *NED* no evidence of disease

Chemotherapy: *MVAC* methotrexate, vinblastine, doxorubicin, and cisplatin (three cycles) after LNUx; *G + CBOCA* gemcitabine and carboplatin (three cycles) after LNUx; *G* gemcitabine (three cycles)

^a^According to Sobin et al. [21]

^b^Interval (days) between LNUx and 14 July or 10 September 2012. If patient died, interval between LNUx and death. In patients who underwent no LNUx, interval between PET/CT and 14 July or 10 September 2012 or death^c^Metastases in the sacral lymph nodes and 4th lumber spine were found 380 days after LNUx

^c^Metastases in the sacral lymph nodes and 4th lumber spine were found 380 days after LNUx

Of the 16 study patients, 13 were choline uptake-positive and the other 3 were uptake-negative in the urothelial lesions (Table 1). In the three choline uptake-negative patients, the SUVmax values were <2.2 at both 10 and 20 min. Except for two inoperable patients and one patient who needed no surgery, all patients underwent LNUx. The specimens obtained by LNUx or autopsy (patient 2) were examined histopathologically, which confirmed that of the 13 choline uptake-positive patients, 11 patients (patients 1, 2, 6 – 12, 14, and 16) had UUT-UC, 1 (patient 3) had malignant lymphoma, and 1 (patient 13) had IgG4-related disease (Table 2). Of the three uptake-negative patients, two (patients 4 and 5) had no malignancies and 1 (patient 15) had UUT-UC (Table 2). Of the 2 patients with no malignancies, one (patient 4) underwent LNUx, and the lesion was confirmed histopathologically to be nonmalignant. The other patient (patient 5) did not undergo LNUx, but no malignancy was diagnosed based on his local and clinical findings. The uptake-negative patient with UUT-UC (patient 15) underwent LNUx and the lesion was confirmed histopathologically to be UUT-UC. Of the 12 patients with UUT-UC, 7 had renal pelvis carcinoma and 5 had ureteral carcinoma (Table 1). In 6 of the 11 choline uptake-positive patients with UUT-UC, the SUVmax at 10 min was higher than at 20 min, and in 5 of these patients the SUVmax at 20 min was higher than at 10 min. There was no statistically significant difference between the SUVmax values of the urothelial lesions at 10 min and those at 20 min.

One patient was false-negative for UUT-UC (patient 15). The other 12 patients with UUT-UC showed choline SUVmax ranging from 2.48 to 7.85 (mean ± SD 4.15 ± 1.73) at 10 min and from 2.83 to 8.08 (3.95 ± 1.54) at 20 min, and a patient with malignant lymphoma in the left ureter (patient 3) also showed choline uptake (SUVmax 2.80 at 10 min and 2.94 at 20 min; Tables 1 and 2). There was incidentally a patient with IgG4-related disease [25, 26] who had a tumorous lesion in the right ureter (patient 13), and the lesion showed weak but definite choline uptake (SUVmax 3.25 at 10 min and 2.64 at 20 min; Tables 1 and 2). The sensitivity of choline PET/CT in the detection of UUT-UC was 92 % (11 of 12 patients). The nonmalignant tumorous ureteral lesion associated with IgG4-related disease with positive uptake was considered false-positive as judged only on the basis of malignancy or nonmalignancy, whereas the other two nonmalignant ureteral lesions in patients 4 and 5 were uptake-negative.

In 5 of the 12 patients with UUT-UC (patients 2, 11, 12, 14 and 16) choline PET/CT also showed definite choline uptake in the sites corresponding to metastases, patients 11 and 14 had metastases only in the regional lymph nodes, and patients 2, 12 and 16 also had distant metastases (Table 3). These metastatic lesions were detected first by choline PET/CT performed in this study. Choline uptake in the metastatic lesions was determined as SUVmax and the size of the lesions was estimated on CT images (Table 3). The SUVmax values in the metastatic lesions were as high as in the primary tumours, ranging from 2.55 to 10.39 (5.75 ± 1.85) at 10 min and from 2.28 to 11.80 (5.45 ± 2.10) at 20 min. 

In 10 of 15 choline uptake-positive metastatic lesions, the SUVmax values at 10 min were higher than those at 20 min, in 4 the SUVmax values at 20 min were higher than those at 10 min, and in 1 the SUVmax values at 10 and 20 min were equal. The SUVmax values at 10 min were significantly higher than those at 20 min (*p* < 0.05). The size of the largest metastatic tumours (41 × 33 × 40 mm, left aortic lymph nodes in patient 16) approximated that of the primary tumour (46 × 45 × 40 mm, right renal pelvis in patient 12). In one inoperable patient (patient 2), metastases were found in the skull, sixth thoracic spine, pelvis, left lung, and the right lower paratracheal and subaortic lymph nodes; in another inoperable patient (patient 16), metastases were found in the left supraclavicular lymph nodes and the left anterior mediastinal lymph nodes as well as in the regional lymph nodes including the left aortic and common iliac lymph nodes (Table 3). The metastatic tumours in the regional and distant lymph nodes were surgically resected (patients 11, 12 and 14) except in two inoperable patients (patients 2 and 16).

**Table 3:** Properties of metastatic lesions

Patient no.	Location	SUVmax		Size (mm)^a^
		10 min	20 min	
2	Right lower paratracheal LN	5.09	4.57	28 × 21 × 14
	Subaortic LN	5.39	4.41	17 × 12 × 10
	Thoracic spine (VI)	5.10	4.71	17 × 15 × 12
	Right iliac bone	9.29	7.60	35 × 33 × 20
	Left lung	2.55	2.28	21 × 17 × 16
	Skull	5.07	4.40	16 × 13 × 16
11	Right internal iliac LN	4.92	5.22	11 × 10 × 9
12	Right renal hilar LN	4.68	3.57	21 × 10 × 12
	Thoracic spine (VI)	6.45	6.01	19 × 16 × 14
	Thoracic spine (XII)	7.36	7.55	36 × 30 × 26
	Right iliac bone	10.39	11.80	28 × 25 × 16
14	Left sacral LN	4.73	5.15	10 × 10 × 10
16	Left aortic LN	5.07	5.46	41 × 33 × 40
	Left common iliac LN	5.85	5.85	30 × 17 × 35
	Left supraclavicular LN	5.91	4.73	20 × 10 × 10
	Left anterior mediastinal LN	4.16	3.82	10 × 10 × 10

*LN* lymph node

^a^Long diameter × short diameter × height

**Fig. 1:**
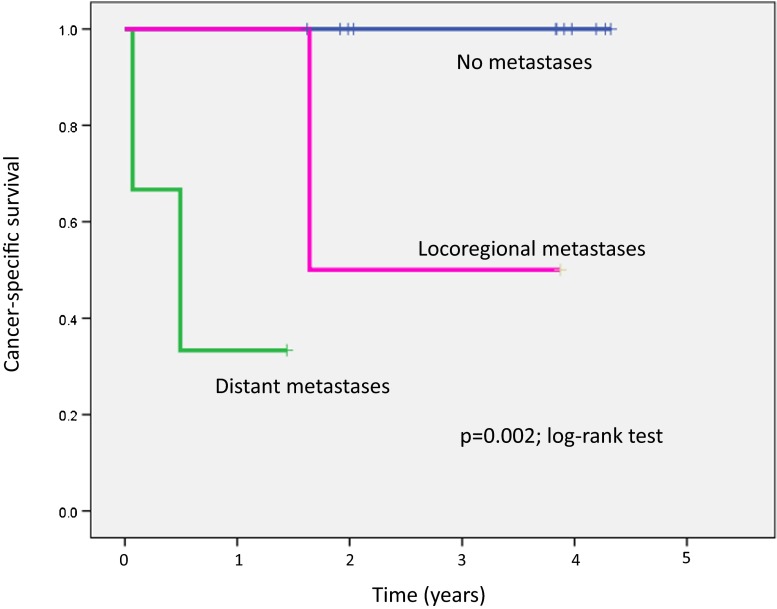
Kaplan-Meier survival curves showing survival in patients with distant metastases (three patients), with metastases only in the regional lymph nodes (two patients), and without metastases (six patients). See the footnotes to Tables 2 and 4

The outcome in all 16 study patients was checked on 14 July or 10 September 2012 (592 – 1,530 days after LNUx; Table 2). The outcomes in patients with distant metastases, with metastases only in the regional lymph nodes, and without metastases are compared in Table 4 and Fig. 1. An inoperable patient (patient 2) died from cancer 25 days after choline PET/CT and another inoperable patient (patient 16) was alive with cancer 510 days after choline PET/CT. Of the other two patients with metastases only in the regional lymph nodes, one (patient 14) died from cancer 600 days after LNUx, and one (patient 11) had no evidence of disease 1,414 days after LNUx. Of seven patients with UUT-UC without metastases, five (patients 1, 7, 8, 9 and 10) had no evidence of disease 1,399 – 1,530 days after LNUx, one (patient 6) died from another cause 725 days after LNUx, and one (patient 15) was alive with cancer 592 days after LNUx, although in this patient metastases in the sacral lymph nodes and fourth lumbar spine were found 380 days after surgery (the footnote relating to patient 15 in Table 2). Patients with malignant lymphoma (patient 3) or with IgG4-related disease (patient 13) had no evidence of disease 1,560 and 743 days after LNUx.

**Table 4:** Outcomes in patients with distant metastases, with metastases only in regional lymph nodes, and without metastases

Patient group	No. of patients	Surgical treatment	Outcome		
			Died from cancer	Alive with cancer	No evidence of disease^g^
With distant metastases	2 (patients 2, 16)	No surgery	1^b^	1^e^	
	1 (patient 12)	LNUx + lymphadenectomy	1^c^		
With metastases only in regional lymph nodes	2 (patients 11, 14)	LNUx + lymphadenectomy	1^d^		1
Without metastases	6 (patients 1, 7, 8, 9, 10, 15)^a^			1^f^	5

*LNUx* laparoscopic nephroureterectomy and partial cystectomy

^a^Seven patients had no metastases, but one died from another cause and was excluded

^b^Died from cancer 25 days after PET/CT

^c^Died from cancer 180 days after LNUx

^d^Died from cancer 600 days after LNUx

^e^Judged 510 days after PET/CT

^f^See the footnote relating to patient 15 in Table 2

^g^Judged 743 – 1,530 days after LNUx

**Fig. 2:**
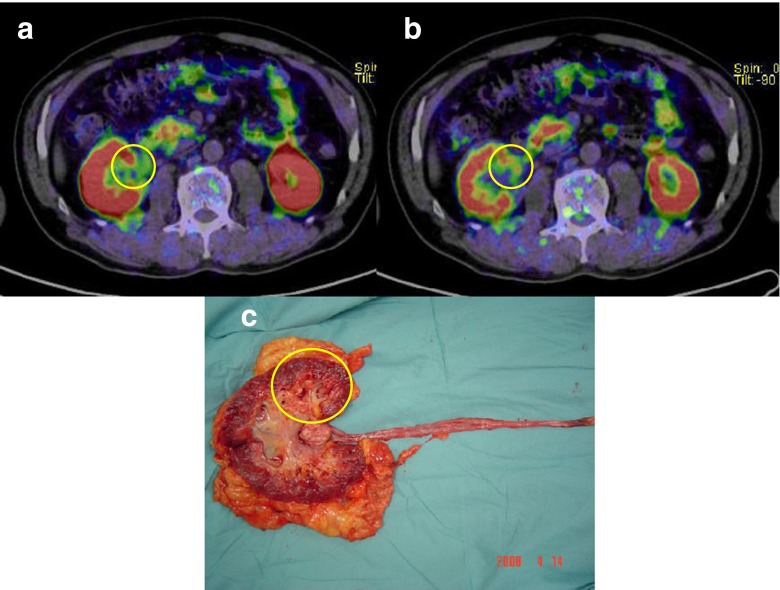
Urothelial carcinoma of the left renal pelvis in patient 1. **a**, **b** Choline PET/CT images of the primary tumour at 10 min (**a**) and 20 min (**b**) after injection. Choline uptake at 10 min is positive but weak (SUVmax 2.48) compared with that at 20 min (SUVmax 3.79). **c** Photograph of the primary tumour after resection. *Circles* tumour sites

**Fig. 3:**
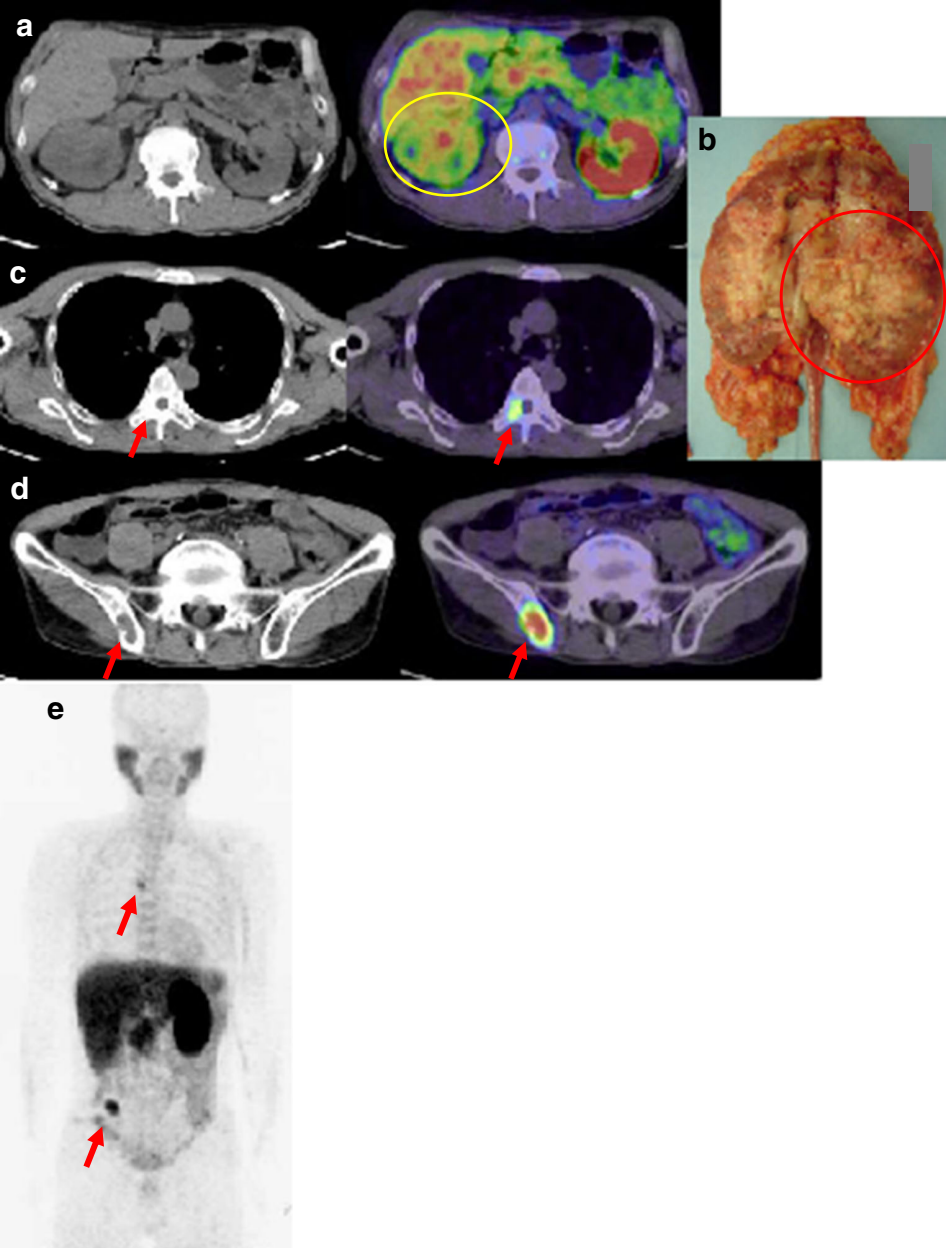
Urothelial carcinoma of the right renal pelvis in patient 12. **a** Choline PET/CT images of the primary tumour at 10 min after injection (SUVmax 7.85). **b** Photograph of the primary tumour after resection. *Circles* tumour sites. **c**, **d** Choline PET/CT images of the metastatic lesions at the 12th thoracic spine (SUVmax 7.36, **c**) and the right iliac bone (SUVmax 10.39, **d**) at 10 min after injection. *Arrows* bone metastases. **e** Maximum intensity projection image. The metastatic lesions in the sixth thoracic spine and the right iliac bone are seen (*arrows*)

**Fig. 4:**
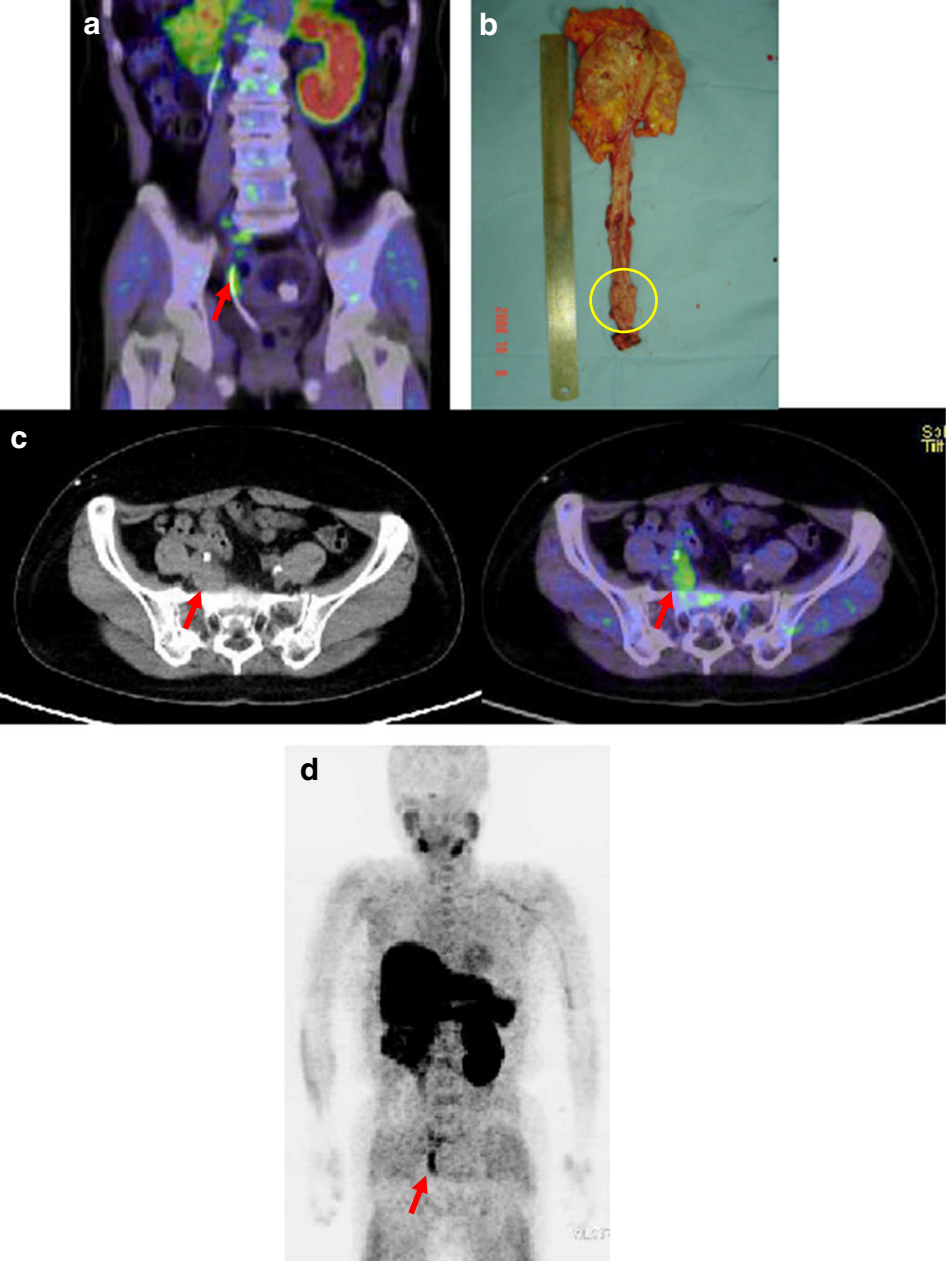
Urothelial carcinoma of the right ureter in patient 11. **a** Choline PET/CT images of the primary tumour at 10 min after injection (SUVmax 6.09). **b** Photograph of the primary tumour after resection. *Circle* tumour site. **c** Choline PET/CT images of the metastatic lesions at the right internal iliac lymph nodes at 10 min after injection (SUVmax 4.92). **d** Maximum intensity projection image. *Arrows* tumour sites. A catheter is inserted into the right ureter (**a**, **d**)

PET/CT images of the primary and metastatic tumours and the photographs of the primary tumours after resection in patients 1, 12 and 11 are shown in Figs. 2, 3 and 4.

## Discussion

^11^C-Choline is a small molecule that after intravenous injection is very quickly integrated into the cell membrane as phosphatidylcholine. It is a marker of membrane metabolism, and is subject to very late urinary excretion, so that the renal pelvis and ureter are free of urinary radioactivity at the time of image acquisition. Nevertheless, there have been no studies so far on the efficacy of choline PET/CT in the detection of the primary tumour and metastases of UUT-UC; this was the principal motive for performing this study.

Of the 16 study patients with suspicion of UUT-UC examined in the present study, in 12 UUT-UC was confirmed. Of the other four, one had malignant lymphoma, one had IgG4-related disease, and two had benign diseases. Of the 12 patients with UUT-TC, 11 (92 %) were choline uptake-positive and the other 1 was false-negative on choline PET/CT. The patient with malignant lymphoma and the patient with IgG4-related disease were uptake-positive, and the two patients with nonmalignant diseases were uptake-negative.

Malignant cells have elevated levels of choline and upregulation of choline kinase activity [27–30]. Studies have been conducted to evaluate the ability and usefulness of choline PET/CT in detecting cancer within the prostate and in differentiating cancer from normal tissue and/or benign disease [16–19]. In summary, these studies indicate that the use of choline PET/CT for initial diagnosis and local staging of prostate cancer cannot be recommended as a first-line screening method in men at high risk of prostate cancer [20]. In contrast, there have been a number of studies demonstrating that choline PET/CT is useful in detecting and staging lymph node metastases of prostate cancer [8–15]. In another study evaluating the ability of choline PET to detect bladder cancer before cystectomy, normal bladder showed little uptake, and primary bladder cancer was visualized in 10 of 18 patients with residual invasive disease in the cystectomy specimen [31].

Of the 12 patients with UUT-UC examined in the present study, choline PET/CT revealed distant metastases in three, metastases only in the regional lymph nodes in two, and no metastases in seven. Both distant metastatic lesions and regional lymph node metastases showed definite choline uptake and similar SUVmax to the values in the primary tumours of UUT-UC. According to the diagnostic findings on choline PET/CT, one of three patients with distant metastases and two patients with metastases only in the regional lymph nodes underwent LNUx and lymphadenectomy, and seven patients without metastases underwent LNUx. As shown in Table 4 and Fig. 1, the outcome in patients with UUT-UC corresponded to the patient classification based on the presence or absence of metastases and locoregional or distant metastases. These results suggest that choline PET/CT is useful in staging as well as in the primary diagnosis of UUT-UC.

 Choline uptakes determined as SUVmax at 10 min were significantly higher than those at 20 min in metastatic tumours of UUT-UC (*p* < 0.05), whereas these was no statistically significant difference between the SUVmax values at 10 min and those at 20 min in the primary tumours of UUT-UC. Although the reason for the difference between the primary and metastatic tumours of UUT-UC is not yet known, the finding suggests that choline uptake takes place earlier in metastatic tumours than in primary tumours of UUT-UC. The results indicate that choline uptake should be determined at both 10 and 20 min after injection for the detection of the primary and metastatic tumours of UUT-UC. Because the number of patients assessed in this pilot study was rather small, confirmation of this conclusion must await further follow-up studies with greater numbers of patients.

Besides UUT-UC, the tumours developed in the ureter of a patient with malignant lymphoma and a patient with IgG4-related disease were choline uptake-positive. IgG4-related disease, which is characterized by elevated serum IgG4 concentrations and tumefaction or tissue infiltration with IgG4-positive plasma cells, is an under-recognized condition about which knowledge is now growing rapidly [25, 26]. The present study demonstrated that inflammatory pseudotumour developed in the ureter of a patient with IgG4-related disease and the tumour showed definite choline uptake. To our knowledge there have been no prior reports of definite choline uptake in tumorous lesions associated with IgG4-related disease.

### Conclusion

The results of the present study showed that choline PET/CT is useful in detecting the primary tumours and metastases of UUT-UC and potentially in providing valuable prognostic information. Besides UUT-UC, the ureteral tumours associated with malignant lymphoma and IgG4-related disease showed definite choline uptake. Follow-up studies with greater numbers of patients are needed to make the conclusions more reliable.
